# CD147 deficiency in T cells prevents thymic involution by inhibiting the EMT process in TECs in the presence of TGFβ

**DOI:** 10.1038/s41423-019-0353-7

**Published:** 2020-01-03

**Authors:** Ruo Chen, Ke Wang, Zhuan Feng, Ming-Yang Zhang, Jiao Wu, Jie-Jie Geng, Zhi-Nan Chen

**Affiliations:** 1 Department of Cell Biology, College of Life Science and Technology, Jinan University, Guangdong, China; 2 National Translational Science Center for Molecular Medicine & Department of Cell Biology, Fourth Military Medical University, Shaanxi, China

**Keywords:** CD147, TGFβ, thymic involution, EMT, CD4-positive T cells, Immune cell death

## Abstract

Thymic involution during aging is a major cause of decreased T-cell production and reduced immunity. Here, we show that the loss of CD147 on T cells prevents thymic senescence, resulting in slowed shrinkage of the thymus with age and increased production of naive T cells. This phenotype is the result of slowing of the epithelial–mesenchymal transition (EMT) process in thymic epithelial cells (TECs), which eventually leads to reduced adipocyte accumulation. In an in vitro coculture system, we found that TGFβ is an important factor in the EMT process in TECs and that it can reduce the expression of E-cadherin through p-Smad2/FoxC2 signaling. Moreover, CD147 on T cells can accelerate the decline in E-cadherin expression by interacting with Annexin A2 on TECs. In the presence of TGFβ, Annexin A2 and E-cadherin colocalize on TECs. However, CD147 on T cells competitively binds to Annexin A2 on TECs, leading to the isolation of E-cadherin. Then, the isolated E-cadherin is easily phosphorylated by phosphorylated Src kinase, the phosphorylation of which was induced by TGFβ, and finally, p-E-cadherin is degraded. Thus, in the thymus, the interaction between T cells and TECs contributes to thymic involution with age. In this study, we illuminate the mechanism underlying the triggering of the EMT process in TECs and show that inhibiting TGFβ and/or CD147 may serve as a strategy to hinder age-related thymic involution.

## Introduction

The thymus is a vital organ for establishing a functional and effective immune system, and it acts by developing immature T cells and exporting mature immune cells into the periphery.^1^ One of the striking paradoxical features of the thymus is that it undergoes profound age-associated atrophy, which results in progressive replacement of the lymphostromal thymic zones with adipocytes, less efficient T-cell development, and decreased naive T-cell emigration.^2–5^ Although thymic decline is of minimal consequence in a healthy individual, the reduced efficacy of the immune system with age has direct etiological linkages with an increase in the incidence of diseases, including opportunistic infections, autoimmune conditions, and cancer.^6^

The mechanisms controlling thymic involution are poorly understood, and several possible mechanisms have been suggested: a blockage in T-cell receptor gene rearrangement, decreased self-peptide MHC molecule levels, and depleted T-cell progenitors.^7,8^ Currently, the investigation to define the intrathymic mechanisms of age-induced thymic atrophy is focused on the loss or disruption of key cross-talk events between developing thymocytes and the supportive thymic stroma, especially thymic epithelial cells (TECs). First, TECs can produce many kinds of cytokines, such as LIF, OSM, SCF, IL-6, and M-CSF, and these molecules exist at significantly higher levels in the aged human thymus than they do in the young human thymus.^9^ These intrathymic cytokines play a crucial role in actively driving thymic involution and inhibiting thymus cell development. Second, TECs are highly proliferative during thymic expansion, and the increased numbers of TECs can enhance, at least temporarily, thymic function.^10–12^ If the proliferation of TECs is blocked, thymic degeneration may be accelerated. The regulation of the cell cycle in TECs can be controlled by Rb family proteins, which also regulate the transcription of Foxn1, an important regulator of TEC differentiation and function.^13^ Third, genetic fate mapping experiments in mice have demonstrated that TECs can undergo the epithelial–mesenchymal transition (EMT) process to become fibroblasts and eventually to transdifferentiate into preadipocytes, suggesting a possible mechanism underlying the generation of adipocytes in the aging thymus.^14,15^ Recently, the transdifferentiation of the TECs during thymic degeneration has been increasingly recognized. Thymic involution appears to be dependent on PPARγ, which drives the transdifferentiation of TECs into adipocytes with age. A systemic decrease in PPARγ activity in mice can prevent thymic involution and elevate T-cell production.^16^ Another report found that caloric restriction can prevent the age-related increase in the expression of EMT regulators, such as forkhead box protein C2 (FoxC2) and fibroblast-specific protein-1 (FSP-1), and inhibit the reduction in the level of lipid-laden thymic fibroblasts.^15^ However, the triggers and progression factors for the EMT process in TECs remain largely unknown.

CD147, also known as Basigin or EMMPRIN, is a highly glycosylated immunoglobulin superfamily protein. Under physiological conditions, this transmembrane protein is widely expressed and plays fundamental roles in various hematopoietic and nonhematopoietic cell lineages.^17–19^ In various cancers, CD147 is overexpressed and associated with a poor prognosis. It is considered a tumor-associated antigen (TAA) because of its intrinsic regulation during tumorigenesis.^20–22^ Usually, CD147 interacts with other proteins to perform important functions.^23–25^ For example, the interaction of CD147 and integrin α5β1 can disrupt hepatocyte polarity to enhance HCC progression by promoting the endocytosis and downregulation of the adhesion molecule E-cadherin and the nuclear translocation of β-catenin.^26^ CD147 and TGFβ form a positive feedback loop to promote EMT and HCC progression.^27^ However, it is unknown whether CD147 interacts with other proteins in the process.

In addition to its critical role in tumorigenesis, CD147 also plays an important role in autoimmune diseases. For example, during the development of RA, CD147 is involved in the activation of memory T cells and the activation of the NLRP3 inflammasomes induced by ACPAs.^28,29^ More importantly, CD147 has also been identified as a signal receiver during immune modulation, especially in T-cell development and activation.^30–32^ In our previous studies, we found that, in young mice, CD147 depletion in thymocytes (CD147^T-KO^) resulted in decreased thymus size, thymic cellularity loss, and diminished T-cell lineage cell numbers, which were accompanied by increases in the number of various lymphocyte populations with innate immunological functions, such as γδ T cells, NKT-like cells, and NK-like cells.^33^ In another interesting finding, the size and weight of the thymus in CD147^T-KO^ mice decreased more slowly than did those in wild-type mice during aging. This result indicates that CD147 on T cells may be involved in thymic involution.

In our study, we investigated whether CD147 on T cells influences thymic involution and the underlying mechanism. We found that the loss of CD147 on T cells can prevent thymic involution by limiting the EMT process in TECs. In vitro, TGFβ is an important factor that triggers the EMT process in TECs through the p-Smad2/FoxC2 signaling pathway, and the interaction of CD147 on T cells with Annexin A2 on TECs accelerates the EMT process in TECs via the Src/p-E-cadherin signaling pathway. Our study is the first to reveal the mechanism that triggers the EMT process in TECs and to show that the interaction between T cells and TECs promotes this process. Therefore, the inhibition of TGFβ and/or CD147 may serve as a strategy to hinder age-related thymic involution.

## Results

### Thymic involution is relatively slow in CD147^T-KO^ mice during aging

**Fig. 1:**
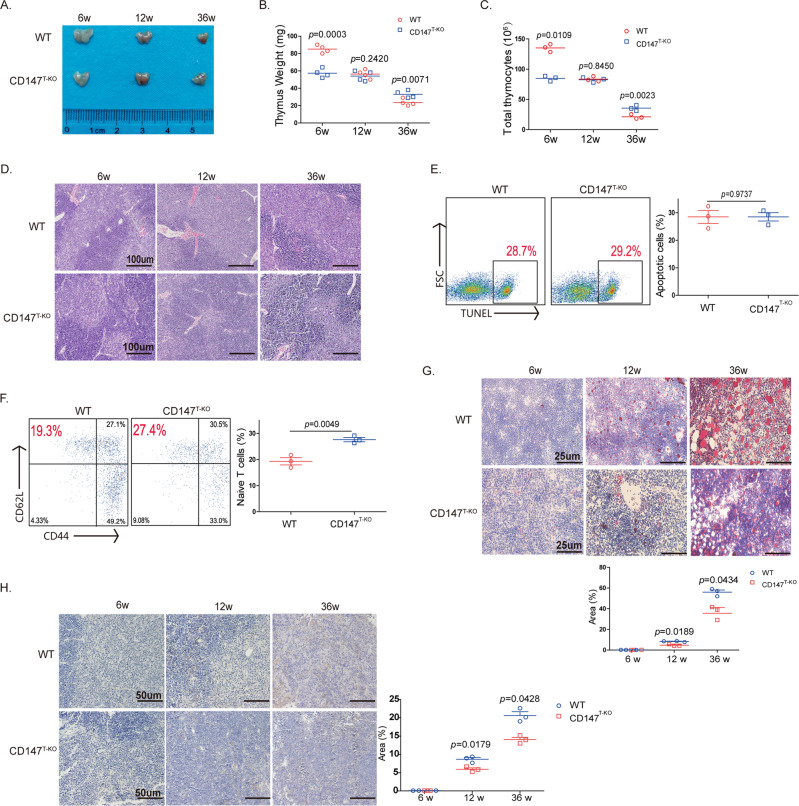
Thymic involution is relatively slow in CD147^T-KO^ mice during aging. **a** Photograph of thymuses isolated from the CD147^T-KO^ mice and wild-type mice at 6, 12, and 36 weeks. **b** Weights of the thymuses isolated from CD147^T-KO^ mice and wild-type mice at 6, 12, and 36 weeks. **c** Total thymocytes in the CD147^T-KO^ mice and wild-type mice at 6, 12, and 36 weeks. **d** Hematoxylin-eosin stained thymuses isolated from the CD147^T-KO^ mice and wild-type mice at 6, 12, and 36 weeks (100×). **e** TUNEL staining showing apoptosis in the DP cells at 12 weeks. **f** Flow cytometry analysis of the naïve T cells in blood collected from the CD147^T-KO^ mice and wild-type mice at 36 weeks. **g** Oil red O staining showing lipid accumulation in the thymus of the CD147^T-KO^ mice and wild-type mice at 6, 12, and 36 weeks (400×). **h** UCP-1 expression in the thymus of the CD147^T-KO^ mice and wild-type mice at 6, 12, and 36 weeks (200×). The data are representative of three or four experiments.

Previously, we reported that CD147 depletion in T cells resulted in deficient thymic development and that both the size and the weight of the thymus were less in the CD147^T-KO^ mice than they were in the wild-type mice 6 weeks after birth.^33^ Interestingly, with the extension of our observation time, we found that the weight of and T-cell number in the thymus in the CD147^T-KO^ mice and wild-type mice were found to be similar 12 weeks after birth (Fig. 1a–c). Then, between the 12th week and the 36th week, the thymus size in wild-type mice continuously decreased until the it was smaller than that of the CD147^T-KO^ mice (Fig. 1a, b). This change in thymus weight was positively correlated with decreases in thymocyte number, which decreased more rapidly in the control group than it did in the CD147-deficient group (Fig. 1c). Histopathological analysis showed no gross alterations in thymic structure in the CD147^T-KO^ mice compared with that of control mice in the 6th, 12th, and 36th week (Fig. 1d). The results from a flow cytometry analysis revealed that CD147 depletion in T cells inhibited the development of T cells in the thymus, including double-negative (DN) cells and double-positive (DP) cells, in the early weeks but had no influence on the proportions of T-cell subpopulations in the wild-type or CD147^T-KO^ mice in the 12th and 36th week (Sup. Fig. 1A). In addition, TUNEL staining showed that the DP cells had similar apoptosis rates in both the wild-type and CD147^T-KO^ mice at the 12th week (Fig. 1e). Notably, more peripheral CD62L + CD44- naive T cells were found in 36-week-old CD147^T-KO^ mice than were found in 36-week-old wild-type mice (Fig. 1f), which indicated that CD147 depletion in T cells increased fresh naive T-cell output in the aged thymus. Interestingly, at 36 weeks, lipids accumulated in the thymus of the wild-type mice, while CD147^T-KO^ mice showed little lipid accumulation in the thymus, as indicated by oil red O staining (Fig. 1g) and LipidTOX fluorescent staining (Sup. Fig. 1B). Thymic adipose tissue shows “beige” characteristics according to molecular, cellular and metabolic profiling; therefore, so we measured the expression of UCP-1, which is a beige-indicative marker, by immunohistochemistry (IHC) staining. The results showed that UCP-1 expression increased with age, but UCP-1 expression in the thymus of the CD147^T-KO^ mice was significantly lower than that it was in the wild-type mice at 36 weeks (Fig. 1h). Overall, these data show that the loss of CD147 in T cells leads to slower thymic involution in aged mice but does not affect thymic structure, T-cell subpopulation distribution in the aged thymus or apoptosis of DP cells.

### Functional TECs in the thymus of aged CD147^T-KO^ mice have a relatively low incidence of EMT

**Fig. 2:**
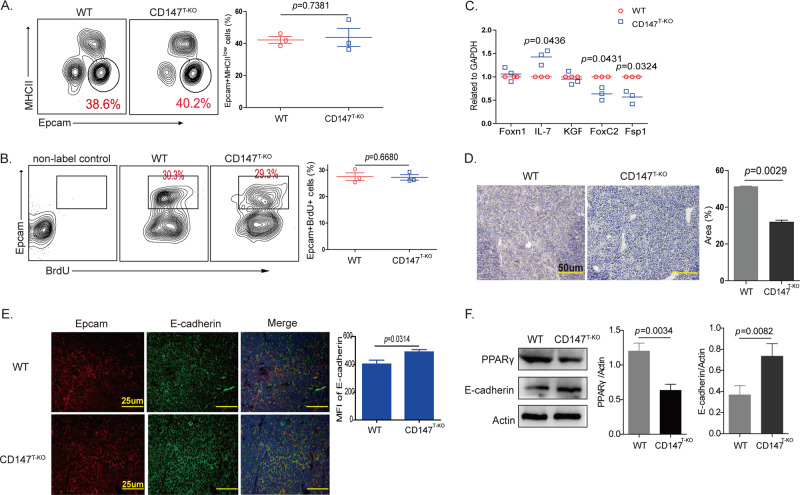
Functional TECs in the thymus of the aged CD147^T-KO^ mice have a relatively low incidence of EMT. **a** Flow cytometry was used to analyze Epcam and MHCII staining of the TECs. **b** TEC proliferation was evaluated using BrdU assays. **c** Real-time PCR was performed to analyze the expression of Foxn1, IL-7, KGF, FoxC2, and Fsp-1. **d** IHC shows the PPARγ expression in the thymus at 12 weeks (200×). **e** Immunofluorescence staining shows E-cadherin expression in the thymus at 12 weeks (400×). **f** Results from the western blot analysis show the expression of PPARγ and E-cadherin in TECs from 12-week-old thymus samples. The data are representative of three experiments.

TECs are a critical component of the thymic environment and perform many of the functions needed to control T-cell differentiation, selection, and involution.^34^ Thus, we wondered whether CD147 deletion in T cells would exert an influence on TECs. First, we detected thymic epithelial progenitors by flow cytometry analysis of cells stained for Epcam and MHCII, markers of putative epithelial progenitors. The results showed that the proportion of Epcam + MHCII^low^ cells was not significantly different in the wild-type or CD147^T-KO^ mice in the 6th week (Fig. 2a). Then, the proliferation of TECs was evaluated with a BrdU assay, and the proportions of BrdU + cells were found to be similar between the two groups of mice (Fig. 2b). The slowed thymic involution in the aged CD147^T-KO^ mice appears to be independent of TEC proliferation and differentiation. Furthermore, we detected the transcription of thymic involution-related genes, such as Foxn1, IL-7, KGF, FoxC2, and Fsp-1, in the 12th week by real-time PCR (Fig. 2c). The expression of Foxn1, an important regulator of TEC differentiation, and KGF, which is involved in the proliferation and differentiation of epithelial cells, showed little difference between the TECs from the CD147^T-KO^ mouse thymus and the TECs from the wild-type mouse thymus, which confirmed that the loss of CD147 in T cells had no influence on the proliferation or differentiation of the TECs. IL-7 is involved in the formation, development, maturation and degradation of the thymus in various ways, and the expression levels of IL-7 decrease in the TECs of aged mice. The increase in IL-7 expression in the TECs from the CD147^T-KO^ mouse thymus confirmed that the loss of CD147 in T cells led to slowed thymic involution in the aged mice. Moreover, we found that the levels of the EMT regulators FoxC2 and Fsp-1 were lower in the TECs of the CD147^T-KO^ mice than in the TECs of the wild-type mice. These results indicated that CD147-deficient T cells may result in EMT inhibition and a decrease in thymic adipogenesis. We detected E-cadherin and PPARγ expression in the thymus from 12-week-old mice by immunofluorescence staining and IHC staining. The expression of PPARγ in the thymus of the CD147^T-KO^ mice was lower than that in the thymus of the wild-type mice (Fig. 2d). The expression of E-cadherin showed the opposite result (Fig. 2e). Then, we collected TECs from 12-week-old mice and detected the expression of PPARγ and E-cadherin using western blotting. The results showed that PPARγ expression was lower and that E-cadherin expression was higher in the CD147^T-KO^ mice (Fig. 2f). These results indicated that the loss of CD147 in T cells can slow thymic involution by changing the transdifferentiation of TECs.

### CD147 expressed on activated CD4 + T cells induces the EMT process in TECs

**Fig. 3:**
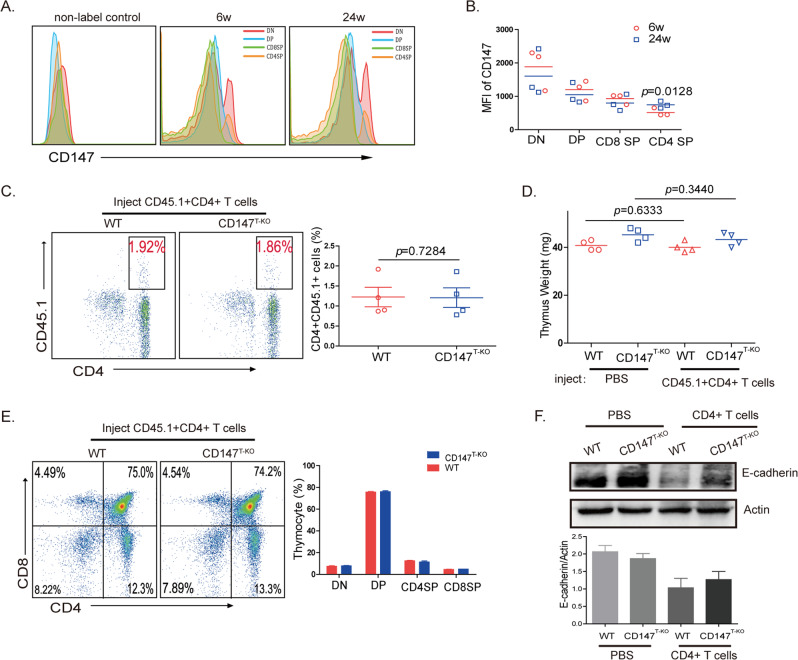
CD147 on activated CD4 + T cells induces EMT in TECs. **a** Flow cytometry analysis results of the CD147 expression level in the T-cell subpopulations isolated from the young and old thymuses. **b** MFI of CD147 in the T-cell subpopulations from the young and old thymuses. **c** Flow cytometry analysis results of CD45.1 + T cells in the thymus 2 months after the mice were injected with activated CD4 + T cells. **d** The weight of the thymus of the mice injected with PBS or activated CD4 + T cells. **e** Flow cytometry analysis results of CD4 + /-CD8 + /- T-cell subpopulation percentages in the thymus of the wild-type and CD147^T-KO^ mice injected with activated CD4 + T cells. **f** Results from the western blot analysis of E-cadherin expression on TECs from the mice injected with PBS or activated CD4 + T cells. The data are representative of three experiments.

According to the results, EMT in TECs is induced by CD147 expressed on T cells. Many kinds of T cells, including DN cells, DP cells, single-positive (SP) cells and activated T cells, are necessary for the development and maturation of the thymus. Therefore, we explored which kinds of T cells expressing CD147 can induce the EMT in TECs. Initially, we hypothesized that T cells with highly expressed CD147 have the ability to promote the EMT in TECs. Flow cytometry results showed that the CD147 expression level was similar between the cells of the young and old thymus (Fig. 3a). However, we found that the mean fluorescence intensity (MFI) of the CD4 + SP cells in the old thymus was higher than it was in the CD4 + SP cells of the young thymus (Fig. 3b). This result is consistent with previous results that showed that a higher number of activated T cells recirculate in the old thymus than are recirculated in the young thymus^35^ and that CD147 is highly expressed on activated T cells.^36^ According to a previous report, coculturing a fetal thymus with activated CD4 + T cells produced a significant inhibitory effect on T-cell development by inducing structural and functional abnormalities in TECs.^37^ These findings suggest that the expression of CD147 on activated CD4 + T cells may induce the EMT process in TECs. Therefore, we sorted CD4 + CD44-CD62L + T cells obtained from the CD45.1 + mice and cultured them in vitro with anti-CD3/CD28 antibodies. After 24 h of stimulation, the expression of CD147 was higher than it was in the naïve T cells (Sup. Fig. 2A). Then, these activated CD4 + T cells (2 × 10^6^ cells/mice) were injected into 12-week-old wild-type and CD147^T-KO^ mice. Two months later, we found similar proportions of CD45.1 + CD4 + T cells in the wild-type and CD147^T-KO^ thymuses (Fig. 3c), which means that the thymuses had a similar capacity to receive mature CD4 + T cells. Compared with the phosphate-buffered saline (PBS)-injected group, the CD4 + T cell-injected group did not exhibit a significant change in thymus weight (Fig. 3d). In addition, flow cytometry analysis results revealed that nominal differences among all the T-cell subpopulations between the wild-type and CD147^T-KO^ thymuses after injection with the CD45.1 + CD4 + T cells (Fig. 3e). However, compared with the CD147^T-KO^ mice in the PBS-injected group, the wild-type and CD147^T-KO^ mice in the T-cell-injected group exhibited decreased expression of the EMT marker E-cadherin on TECs (Fig. 3f). Therefore, we hypothesized that the nonsignificant differences in thymus size (Fig. 3d) may have been attributable to the short observation time. These results suggest that CD147 expressed on activated CD4 + T cells had the capacity to induce EMT.

### TGFβ is an important factor in CD147-induced EMT in the thymus in vitro

**Fig. 4:**
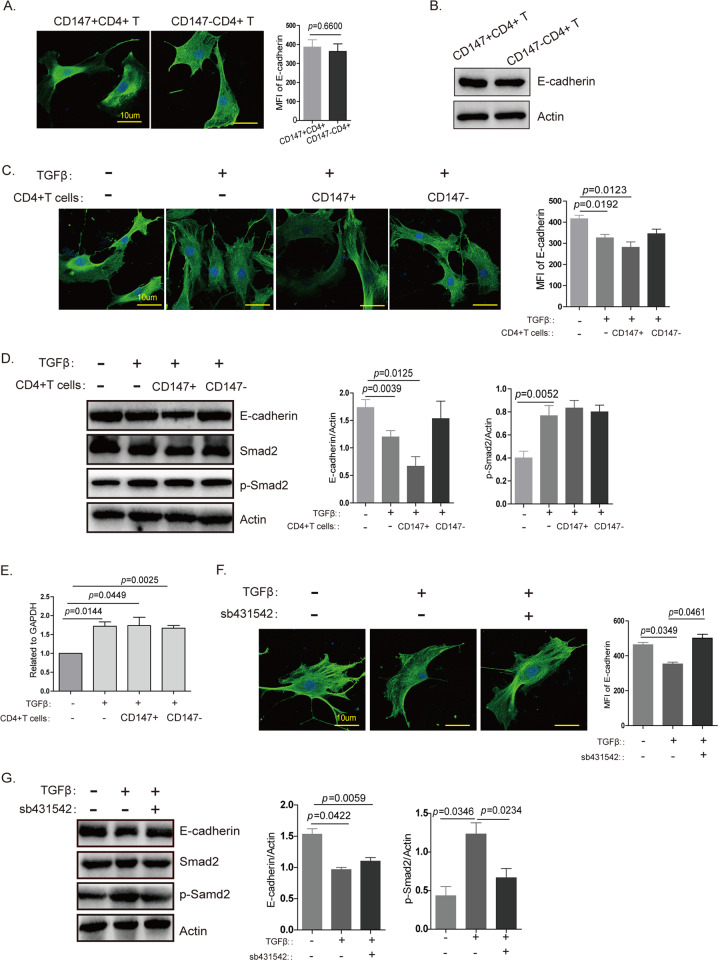
TGFβ is an important factor in CD147-induced EMT in the thymus in vitro. **a** Immunofluorescence staining shows E-cadherin expression on TECs cocultured with CD147 + CD4 + or CD147-CD4 + SP cells without TGFβ. **b** Western blot analysis results show E-cadherin expression in TECs cocultured with CD147 + CD4 + or CD147-CD4 + SP cells without TGFβ. **c** Immunofluorescence staining shows E-cadherin expression on TECs cocultured with CD147 + CD4 + or CD147-CD4 + SP cells in the presence of TGFβ. **d** Western blot analysis results show E-cadherin, Smad2 and p-Smad2 expression in TECs cocultured with CD147 + CD4 + or CD147-CD4 + SP cells in the presence of TGFβ. **e** Real-time PCR was performed to analyze the expression of FoxC2 in TECs cocultured with CD147 + CD4 + or CD147-CD4 + SP cells in the presence of TGFβ. **f** Immunofluorescence staining shows E-cadherin expression on TECs cocultured with CD147 + CD4 + cells in the presence of TGFβ or a TGFβ inhibitor. **g** Western blotting shows E-cadherin, Smad2 and p-Smad2 expression in TECs cocultured with CD147 + CD4 + cells in the presence of TGFβ or a TGFβ inhibitor. These images were obtained through the oil immersion lens of a confocal microscope (1000×), and the data are representative of three experiments.

To clarify the mechanism underlying how CD147 expression on T cells mediates thymic involution, we established a simple and convenient in vitro method. We isolated Epcam + TECs using flow cytometry and cocultured CD147 + CD4 + or CD147-CD4 + T cells with TECs. However, the results indicated little difference in E-cadherin expression (Fig. 4a, b). This result suggests that the interaction between T cells and TECs did not induce the EMT process in TECs. In tumors, CD147 can induce EMT in the presence of TGFβ,^22^ and according to a previous report, TGFβ may be an important factor in thymic involution.^4^ Therefore, we added TGFβ into the coculture system, and the results showed that the expression of E-cadherin decreased slightly in TECs (Fig. 4c, d). When cocultured with activated CD147 + CD4 + T cells, the TECs exhibited a more obvious decrease in the expression of E-cadherin, but the CD147-CD4 + group showed no changes in E-cadherin expression (Fig. 4c, d). During the EMT, TGFβ transduces intracellular signals through a tetrameric complex of receptor-mediated Smad2 phosphorylation.^38^ In our study, TGFβ induced an increase in p-Smad2 expression (Fig. 4d), which in turn promoted FoxC2 expression (Fig. 4e). When a TGFβ inhibitor was used to inhibit TGFβ/p-Smad2 signaling, E-cadherin expression increased (Fig. 4f, g). Therefore, our study results suggest that TGFβ induces the EMT in TECs through the p-Smad2/FoxC2 signaling pathway and that TGFβ is an important factor in CD147-induced EMT in the thymus in vitro.

### EMT in TECs can be induced by the interaction between Annexin A2 on TECs and CD147 on T cells in the presence of TGFβ

**Fig. 5:**
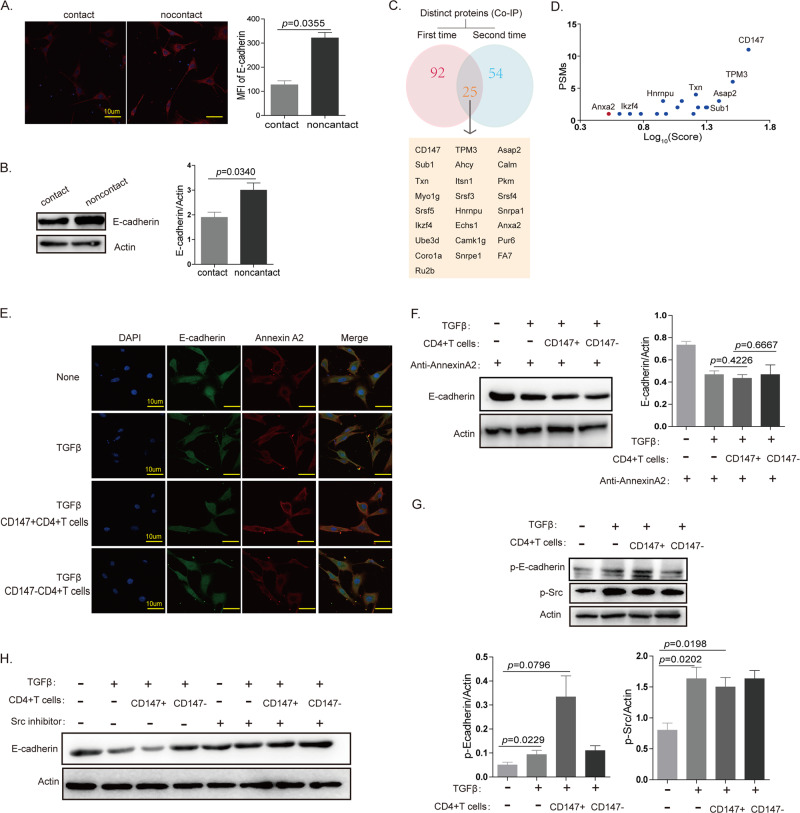
EMT of TECs can be induced by the interaction between Annexin A2 on TECs and CD147 on T cells in the presence of TGFβ. **a** IF staining analysis of E-cadherin expression on TECs cocultured with CD147 + CD4 + cells in a contact or noncontact system in the presence of TGFβ (600×). **b** Western blot analysis results of E-cadherin expression on TECs cocultured with CD147 + CD4 + cells in a contact or noncontact system in the presence of TGFβ. **c** Mass spectrometry analysis results of the co-IP-obtained proteins. **d** PSMs and Log_10_ (score) analysis results of the co-IP-obtained proteins. **e** The locations of Annexin A2 and E-cadherin in TECs, as determined using confocal microscopy (600×). **f** Western blot analysis results of E-cadherin expression in TECs cocultured with CD147 + CD4 + or CD147-CD4 + cells in the presence of TGFβ and anti-Annexin A2. **g** Western blot analysis results of p-E-cadherin and p-Src expression in TECs cocultured with CD147 + CD4 + or CD147-CD4 + cells in the presence of TGFβ. **h** Western blot analysis results of E-cadherin expression in TECs cocultured with CD147 + CD4 + or CD147-CD4 + cells in the presence of TGFβ and a Src inhibitor. The data in **c** and **d** are representative of two experiments, and the data in the other panels are representative of three experiments.

In the presence of TGFβ, the expression of p-Smad2 and FoxC2 showed no difference between the coculture groups of CD147 + /-CD4 + T cells and TECs, while E-cadherin expression on TECs decreased significantly in the group of cocultured CD147 + CD4 + T cells and TECs (Fig. 4c–e). These results suggest that CD147 on T cells induces a decrease in E-cadherin expression in addition to affecting p-Smad2 expression. In the presence of TGFβ, CD147 + CD4 + T cells and TECs were cocultured in contact and noncontact systems. The results from immunofluorescence staining and western blotting showed that E-cadherin expression on TECs was higher in the noncontact system (Fig. 5a, b). Considering that CD147 must interact with other proteins to function effectively, we wondered whether CD147 on T cells interacted with other proteins on TECs to promote EMT. We collected enough thymocyte protein from the mouse thymus to carry out a coimmunoprecipitation (Co-IP) assay. The results indicated that Annexin A2 may interact with CD147 during thymic involution (Fig. 5c, d). Annexin A2 is involved in the EMT process in cancer and colocalizes with E-cadherin in the presence of TGFβ.^39^ Immunofluorescence staining showed that Annexin A2 and E-cadherin were uniformly distributed on TECs in the presence of TGFβ; however, Annexin A2 tended to aggregate on the membrane for enhanced binding to CD147 on T cells when TECs were cocultured with CD4 + CD147 + T cells, and in this condition, the expression of E-cadherin was decreased (Fig. 5e and Sup. Fig. 3A). These results revealed that the interaction of CD147 on T cells and Annexin A2 on TECs may play an important role in EMT of TECs. To confirm the interaction between CD147 and Annexin A2, we added Annexin A2 antibody into the coculture system, and the results from the western blot analysis showed that E-cadherin expression did not decrease significantly on TECs cocultured with CD4 + CD147 + T cells (Fig. 5f). TGFβ can induce the phosphorylation of Src kinase, and E-cadherin can be phosphorylated by Src kinase. Therefore, we measured the expression levels of p-Src and p-E-cadherin and found that TGFβ induced the phosphorylation of Src kinase and that the phosphorylation of E-cadherin was increased in the CD147 + CD4 + T cell and TEC coculture system and led to the degradation of E-cadherin (Fig. 5g). When an Src inhibitor was added into the cocultured system, the expression of E-cadherin rebounded (Fig. 5h). Our data indicated that the interaction of CD147 on CD4 + T cells with Annexin A2 on TECs promoted E-cadherin dissociation from Annexin A2 in the presence of TGFβ; the isolated E-cadherin protein was then easily phosphorylated by Src kinase and degraded. The degradation of E-cadherin led to the EMT process in TECs and to thymic involution.

**Fig. 6:**
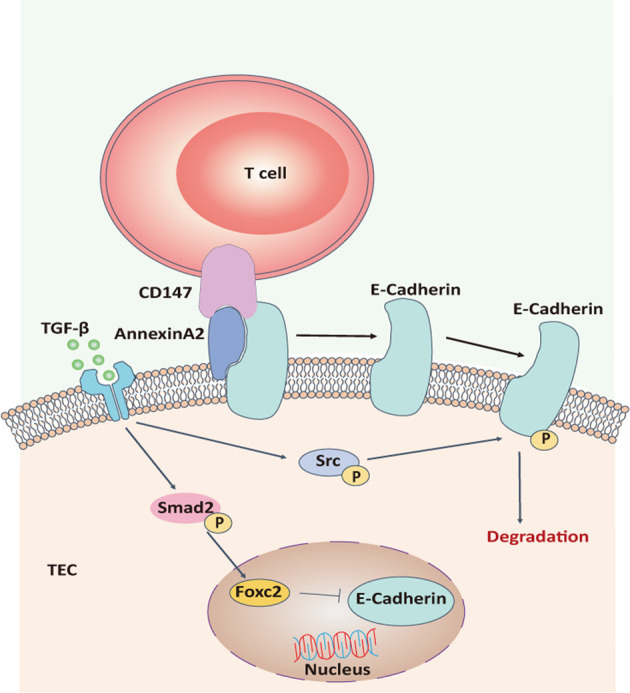
The proposed molecular network for age-related thymic involution. TGFβ is an important factor that triggers the EMT process in TECs through the p-Smad2/FoxC2 signaling pathway, and the interaction of CD147 on T cells with Annexin A2 on TECs accelerates the EMT process in TECs via the Src/p-E-cadherin signaling pathway.

In our study, we confirmed that the loss of CD147 on T cells can prevent thymic involution by limiting the EMT process in TECs. TGFβ is an important factor that triggers the EMT process in TECs through p-Smad2/FoxC2 signaling, and the interaction of CD147 on T cells with Annexin A2 on TECs accelerates the EMT process in TECs via the Src/p-E-cadherin signaling pathway. Therefore, the inhibition of TGFβ and/or CD147 may serve as a strategy to hinder age-related thymic involution (Fig. 6).

## Discussion

In this study, we found that CD147 on T cells can promote thymic involution by mediating the EMT process in TECs, although it had been reported that CD147 on T cells can inhibit thymic development by changing the direction of T-cell development. Therefore, it is reasonable that CD147 plays different roles in different thymic stages in the thymus.

Thymic involution is a dynamic process that is characterized by abnormal thymic structure, changes in the cellularity of intrathymic T-cell subsets, and progressive replacement of TECs with adipocytes, resulting in a steady decline in the production of naïve T cells.^40,41^ Knocking out CD147 in T cells slows the accumulation of beige adipocytes in the thymus, which is accompanied by increased naive T-cell output, indicating that thymic degeneration has slowed. Increasing evidence indicates that TECs can transdifferentiate into preadipocytes during age-related thymic involution.^42,43^ It has been reported that the expression of E-cadherin, an excellent molecular marker of EMT, is downregulated in the TECs of aged mice,^44^ indicating the possible involvement of the EMT process in age-related thymic involution. Our study confirmed that the loss of CD147 expression in T cells resulted in a slow decrease in E-cadherin expression in TECs; when activated CD4 + T cells with normal expression levels of CD147 were injected into CD147^T-KO^ mice, E-cadherin expression on TECs was decreased. These results suggest that CD147 on T cells is closely related to the induction of the EMT process in TECs.

It is known that T cells need to interact with TECs to promote thymic development. For example, N1-expressing thymic progenitors interact with DL4-expressing TECs to suppress B lineage potential and to induce the first steps of intrathymic T-cell development.^27^ However, it is unknown whether the interaction between these T cells and TECs is necessary for thymic involution. When TECs and CD4 + CD147+/− T cells were cocultured in vitro, the EMT process was not induced in the TECs. It is possible that there are many other factors involved in this process in vivo. TGFβ, a potent inducer of EMT, is expressed in all cells of the embryonic thymus, suggesting a critical role for TGFβ in thymic development.^45^ Subsequent studies have shown that TGFβ mRNA levels are dramatically increased in the aged human thymus^46^, but only transiently increased in the aged mouse thymus.^47^ Additionally, TEC-specific depletion of the TGFβ type II receptor in mice increases the size of the medullary TEC compartment, promotes negative selection and the development of Hassall’s corpuscles, and impedes the progression of thymic senescence.^47–49^ These findings suggest that TGFβ signaling positively participates in age-related thymic involution. Therefore, we added TGFβ to our established in vitro coculture system, and as we expected, TGFβ induced decreased expression of E-cadherin. FoxC2, a member of the forkhead transcription factor family, is expressed in various embryonic tissues and is required for the organogenesis of mesodermal tissues, including bone tissue, vascular tissue, and lymphatic tissue.^50–52^ Elevated expression of FoxC2 is also observed in the aged thymus and may be associated with age-related deterioration effects on the thymic stromal cell microenvironment.^15^ In addition, TGFβ is able to activate FoxC2 by phosphorylating Smad2 to promote the expression of mesenchymal proteins.^53,54^ In the EMT, TGFβ transduces intracellular signaling through a tetrameric complex with its receptor to phosphorylate Smad2. After being phosphorylated, Smad2 combines with Smad4 and then translocates into the nucleus to activate FoxC2. In our study, we added TGFβ to our coculture system, and the results showed that the expression of E-cadherin decreased slightly in TECs and that TGFβ induced an increase in the level of p-Smad2, which in turn promoted the expression of FoxC2. E-cadherin expression increased when a TGFβ inhibitor was used to inhibit TGFβ/p-Smad2 signaling. In the presence of TGFβ, the expression of E-cadherin on TECs was decreased more obviously in the CD147 + CD4 + T cell and TEC cocultures than in the CD147-CD4 + T cell and TEC cocultures, in which no change in E-cadherin expression was found. Therefore, we suggest that TGFβ induces the EMT in TECs through the p-Smad2/FoxC2 signaling pathway and that TGFβ is an important factor for the CD147-induced EMT in the thymus. CD147 is highly expressed in thymic DN cells, decreased in mature resting T cells, and increased again when T cells are activated. With increasing age, mature activated T cells in the thymus, especially CD4 + T cells, can accelerate the aging of the thymus. TGFβ is upregulated in the aging human and mouse thymus, suggesting a positive role for TGFβ signaling in age-related thymic involution. TGFβ and CD147 on T cells accelerate the age-thymus involution synergistically.

In the presence of TGFβ, the expression of p-Smad2 and FoxC2 was not different in the coculture group composed of CD147 + /−CD4 + T cells and TECs, while E-cadherin expression on TECs decreased significantly in the CD147 + CD4 + T cell and TEC coculture group. These results suggest that CD147 on T cells functions through a distinct mechanism to induce a decrease in E-cadherin expression in TECs, in addition to the p-Smad2/FoxC2 signaling pathway. In the presence of TGFβ, CD147 + CD4 + T cells and TECs were cocultured in contact and noncontact systems. The results from the western blot analysis showed that E-cadherin expression on TECs was higher in the noncontact system. Considering that CD147 must interact with proteins to function effectively, we wondered whether CD147 on T cells interacts with other proteins on TECs to promote EMT. To date, many proteins interacting with CD147 have been identified, and Annexin A2 is one of them. Annexin A2 can directly bind to CD147 to promote cell movement.^55^ According to our mass spectrometry results, Annexin A2 on TECs was most likely to interact with CD147 on T cells. Importantly, it has been reported that Annexin A2 and E-cadherin colocalize at cellular junctions after TGFβ activation in colorectal cancer cells.^39^ In our study, we also found that TGFβ could induce the colocalization of Annexin A2 and E-cadherin in TECs. However, when T cells were included in this scenario, CD147 on the T cells competitively bound to Annexin A2 on the TECs, leaving E-cadherin isolated. The isolated E-cadherin was easily phosphorylated by Src kinase and then degraded. The degradation of E-cadherin led to the EMT process in TECs and to thymic involution. Our data indicate that TGFβ is an important trigger of thymic involution and that the interaction between CD147 on T cells and Annexin A2 on TECs can accelerate this process.

In this study, we were the first to uncover the mechanism of the induction of the EMT process in TECs involved in thymic involution. TGFβ may be an important factor that triggers EMT in TECs, and the interaction between CD147 on T cells and Annexin A2 on TECs can accelerate the EMT process. This study suggests that inhibiting TGFβ and/or CD147 may serve as a strategy to hinder age-related thymic involution.

## Materials and methods

### Animals

Conditional CD147^T-KO^-knockout mice were developed according to a standard gene-targeting approach using embryonic stem (ES) cells and were used in previous experiments. The CD147^T-KO^ mice were on a mixed C57BL/6J and 129S5 genetic background. Normal female C57BL/6 mice were purchased from the Laboratory Animal Center of Fourth Military Medical University. All animal experiments were approved by the Laboratory Animal Ethics Committee of Fourth Military Medical University (animal ethics approval No: KY20183369-1) and performed in strict accordance with good animal practices as defined by the relevant national animal welfare bodies. All mice were raised in an SPF-level environment.

### Flow cytometry and cell sorting

Cells from the thymus were mechanically disrupted, and the red blood cells were removed by incubation in a red cell lysis buffer (TIANGEN, China) for 10 min on ice. Cells cultured in vitro were collected and washed with PBS with 1% BSA prior to antibody labeling. The following antibodies were used: PerCP-conjugated anti-CD3e (553067, BD, USA), FITC-conjugated anti-CD4 (100405, BioLegend, UK), PE-conjugated anti-CD8a (553033, BD, USA), PE-conjugated anti-CD62L (553151, BD, USA), PE-Cy7-conjugated anti-CD44 (560569, BD, USA), PE-conjugated anti-CD147 (562676, BD, USA), PerCP/Cy5.5-conjugated anti-CD45.1 (110728, BioLegend, UK), anti-P63 (ab110038, Abcam, UK), anti-Epcam (ab71916, Abcam, UK), and anti-BrdU (ab6326, Abcam, UK). Some cells were incubated with fluorescently labeled antibodies for 30 min at 4 °C before being washed, and some cells were incubated with unlabeled antibodies for 1 h at 4 °C, washed, and then incubated with fluorescently labeled secondary antibodies for 30 min at 4 °C before being washed again. For intracellular staining, cell permeabilization was performed using a Cytofix/Cytoperm fixation/permeabilization solution kit (BD Biosciences, USA) for 30 min at 4 °C. Data acquisition was performed using a FACSCalibur flow cytometer (BD Biosciences) with dead cell exclusion based on their scattering profile or by propidium iodide (PI) incorporation. The analysis was performed using FlowJo software (Tree Star). Sorting was performed using a FACS Aria cell sorter (BD Biosciences).

### BrdU uptake in vivo

Mice were intraperitoneally injected with BrdU at 100 mg/kg, and after 12 h, thymus tissue was removed for detection of cell proliferation.

### RNA extraction and real-time quantitative PCR analysis

Total RNA was extracted from the TECs using a Total RNA Kit II (Omega Bio-tek, USA) according to the manufacturer’s instructions. Complementary DNA (cDNA) was synthesized from 1 μg of total RNA using an RNA reverse transcriptase kit (TaKaRa, Japan). Real-time quantitative PCR was performed using an ABI PRISM 7000 sequence detection system (Applied Biosystems), and a TaKaRa SYBR Premix Ex Taq II Kit was used for amplification according to the manufacturer’s instructions. The cDNA input was standardized, and 40 cycles of PCR were performed.

### Hematoxylin and eosin staining

Formalin-fixed paraffin-embedded thymus sections were deparaffinized in xylene and alcohol. The slides were then counterstained with hematoxylin for 15 min and eosin for 10 min. Then, the tissue sections were dehydrated and mounted in a resinous medium.

### IHC staining

Antigen retrieval with 10 μmol/L citrate buffer at pH 6.0 was performed on formalin-fixed paraffin-embedded thymus sections. Anti-UCP-1 (sc-518024, Santa Cruz, USA) and anti-PPARγ (16643-1-AP, Proteintech, USA) were selected as the primary antibodies to incubate the slices.

### TUNEL assay

Cell apoptosis in the thymus was detected using the FragEL^TM^ DNA fragmentation detection kit (QIA39; Merck Millipore, Germany) according to the manufacturer’s instructions. The ratios of positive cell counts vs. total cell counts were determined by flow cytometry.

### Immunofluorescence staining

Immunofluorescence staining was performed using paraffin-embedded thymus sections. The paraffin-embedded sections were dewaxed, and antigen retrieval was performed with 10 μmol/L citrate buffer at pH 6.0. The TECs were cultured in small dishes and fixed with 4% paraformaldehyde. The paraffin-embedded sections and TECs were permeabilized with 0.2% Triton X-100, blocked with BSA, and then stained with anti-PPARγ (16643–1-AP, Proteintech, USA), anti-E-cadherin (EM0502, HuaBio, China), anti-Epcam (ab71916, Abcam, UK) or anti-Annexin A2 (11256–1-AP, Proteintech, USA) antibodies. After incubation with fluorescently labeled secondary antibodies, the sections and dishes were examined by light microscopy.

### TECs cocultured with T cells in vitro

The thymus from C57BL/6 mice (6 weeks) was digested until it was a single-cell suspension, and the Epcam + cells were sorted by flow cytometry. Then, the sorted cells were cultured for one day and then cocultured with CD147 + CD4 + or CD147-CD4 + cells in the presence or absence of TGFβ. After 3 days, the T cells were washed, and the TECs were collected for follow-up experiments.

### E-cadherin phosphorylation assays

The cells were washed with PBS and lysed with an IP lysis/wash buffer with a protease inhibitor and phosphatase inhibitor (Roche, Switzerland) on ice for 30 min. The cleared lysates were quantified, and an equal amount of each lysate was used for immunoprecipitation with a Pierce® Crosslink IP kit (26147, Thermo Fisher Scientific, USA), including Pierce protein A/G Plus agarose, on which an anti-E-cadherin antibody had been previously bound (20874–1-AP, Proteintech, USA). Precleared lysate was added to the antibody-cross-linked resin in a column, which was shaken overnight at 4 °C. The resins were washed with IP lysis/wash buffer, Tris-buffered saline (TBS) and conditioning buffer, and the samples were eluted with elution buffer. The elution fractions were further evaluated on an sodium dodecyl sulfate–polyacrylamide gel electrophoresis (SDS-PAGE) gel. Subsequent immunoblotting was performed using a pan anti-phosphotyrosine antibody (ab10321, Abcam, UK).

### Western blot analyses

The freshly isolated TECs were immediately homogenized in a protein lysis buffer containing phenylmethylsulfonyl fluoride, protease inhibitors, and phosphatase inhibitors (Roche, Switzerland). A BCA protein assay kit (Thermo Fisher Scientific, USA) was used to quantify the amounts of different proteins. After boiling for 10 min, equal amounts of the protein lysis buffer samples were loaded and separated by 10% SDS-PAGE and then transferred to polyvinylidene fluoride membranes (Millipore, USA). After blocking with 5% nonfat milk for 1 h, the membranes were incubated with an anti-E-cadherin antibody (EM0502, HUABIO, China), anti-Smad2 antibody (5339s, Cell Signaling Technology, USA), anti-p-Smad2 antibody (ET1612–32, HUABIO, China) or anti-p-Src antibody (orb99267, Biorbyt, UK) at 4 °C overnight. After several washes, the membranes were incubated with HRP-conjugated secondary antibodies at room temperature for 1 h and then visualized using ECL reagents (Amersham Bioscience).

### Co-IP and mass spectrometry

The thymocytes from C57BL/6 mice (older than 12 weeks) were treated with an IP lysis/wash buffer, and co-IP was performed using a Pierce® Co-IP Kit (26149, Thermo Fisher Scientific, USA) according to the manufacturer’s protocol. The eluted samples were loaded onto a 10% SDS-PAGE gel. After silver staining (Pierce Silver Stain), the trypsin-digested protein samples were analyzed with a mass spectrometer (Model LTQ, Thermo Fisher Scientific). CD147 antibody (11989–1-AP, Proteintech, USA) was used for the IP assay of CD147, and IgG antibody was used as a negative control. After obtaining the mass spectrometry data, the proteins obtained in the IgG group were removed from the CD147 group, and the target protein that interacted with CD147 was found in two independent experiments.

### Statistical analysis

GraphPad Prism software was used to analyze the data. Means, SD values and probability values (*p*) are presented in some figures. Analysis of variances were used to compare more than two groups, and paired *t*-tests was used to compare findings between two groups. *p < 0.05 was considered significant.

## Supplementary information

Supplemental Figure 1

Supplemental Figure 2

Supplemental Figure 3
