# Supplementary material for: CD147 deficiency in T cells prevents thymic involution by inhibiting the EMT process in TECs in the presence of TGFβ
Source: Cell Mol Immunol. 2020 Jan 3;18(1):171–81. doi: 10.1038/s41423-019-0353-7 (PMC7853129; doi:10.1038/s41423-019-0353-7)
Supplement: Supplementary file 1 — Supplemental Figure 1 [file 41423_2019_353_MOESM1_ESM.pdf]

Supplementary Figure1

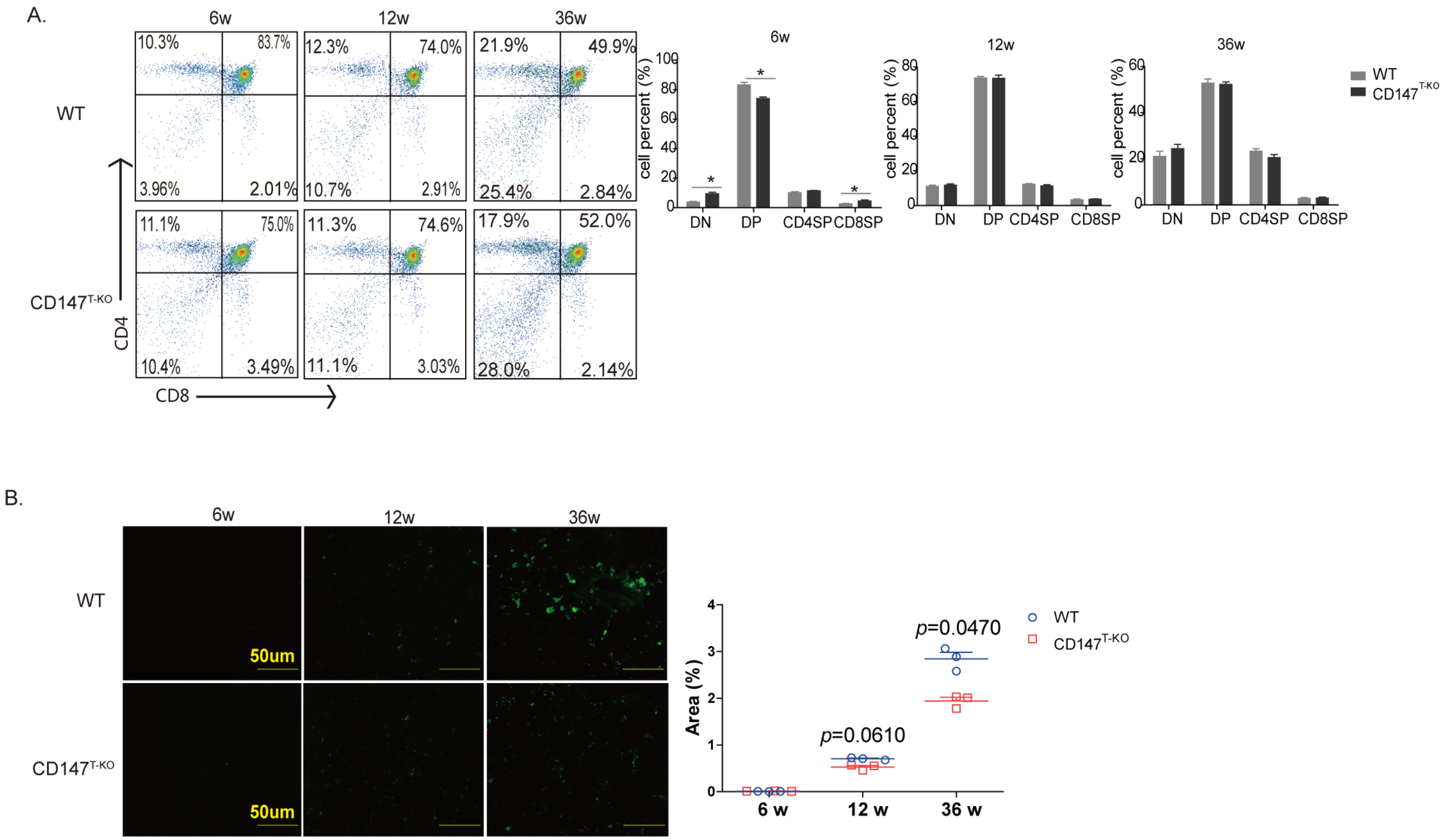

Sup.Fig1. A. Flow cytometry analysis of T cells subpopulations at 6, 12, and 36 weeks. B. LipidTox fluorescent staining showing lipid accumulation in the thymus of CD147$^{T\text{-}KO}$ mice and wild-type mice at 6, 12, and 36 weeks. The data are representative of 3 experiments.
