# Supplementary figures and images for: CD147 deficiency in T cells prevents thymic involution by inhibiting the EMT process in TECs in the presence of TGFβ
Source: Cell Mol Immunol. 2020 Jan 3;18(1):171–81. doi: 10.1038/s41423-019-0353-7 (PMC7853129; doi:10.1038/s41423-019-0353-7)

## Supplementary Figure2

A.

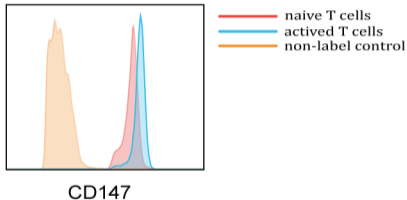

**Sup.Fig2.** A. The expression of CD147 was detected by flow cytometry.

Supplement: Supplementary file 2 — Supplemental Figure 2 [file 41423_2019_353_MOESM2_ESM.pdf]

### Supplementary Figure3

A.

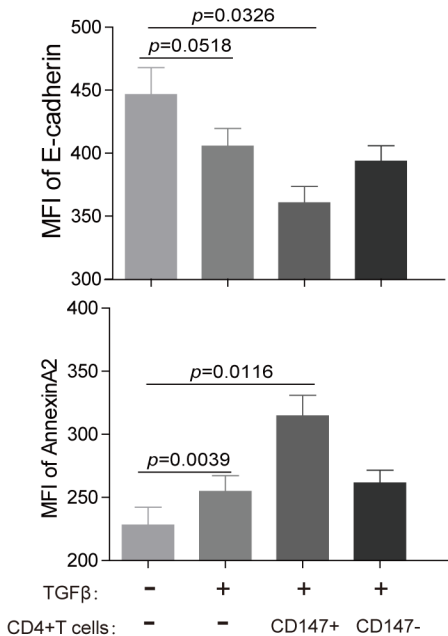

**Sup.Fig3. A.** The statistic analysis of E-cadherin and AnnexinA2.

Supplement: Supplementary file 3 — Supplemental Figure 3 [file 41423_2019_353_MOESM3_ESM.pdf]
